# A Bivalent Anthrax–Plague Vaccine That Can Protect against Two Tier-1 Bioterror Pathogens, *Bacillus anthracis* and *Yersinia pestis*

**DOI:** 10.3389/fimmu.2017.00687

**Published:** 2017-06-26

**Authors:** Pan Tao, Marthandan Mahalingam, Jingen Zhu, Mahtab Moayeri, Michelle L. Kirtley, Eric C. Fitts, Jourdan A. Andersson, William S. Lawrence, Stephen H. Leppla, Ashok K. Chopra, Venigalla B. Rao

**Affiliations:** ^1^Department of Biology, The Catholic University of America, Washington, DC, United States; ^2^Microbial Pathogenesis Section, Laboratory of Parasitic Diseases, National Institute of Allergy and Infectious Diseases, National Institutes of Health, Bethesda, MD, United States; ^3^Department of Microbiology and Immunology, University of Texas Medical Branch, Galveston, TX, United States; ^4^Galveston National Laboratory, University of Texas Medical Branch, Galveston, TX, United States; ^5^Institute for Human Infections and Immunity, University of Texas Medical Branch, Galveston, TX, United States; ^6^Sealy Center for Vaccine Development, University of Texas Medical Branch, Galveston, TX, United States; ^7^Center for Biodefense and Emerging Infectious Diseases, University of Texas Medical Branch, Galveston, TX, United States

**Keywords:** biodefense vaccines, bivalent vaccines, plague vaccine, anthrax vaccines, capsular antigen f1, low calcium response V antigen LcrV, protective antigen

## Abstract

Bioterrorism remains as one of the biggest challenges to global security and public health. Since the deadly anthrax attacks of 2001 in the United States, *Bacillus anthracis* and *Yersinia pestis*, the causative agents of anthrax and plague, respectively, gained notoriety and were listed by the CDC as Tier-1 biothreat agents. Currently, there is no Food and Drug Administration-approved vaccine against either of these threats for mass vaccination to protect general public, let alone a bivalent vaccine. Here, we report the development of a single recombinant vaccine, a triple antigen consisting of all three target antigens, F1 and V from *Y. pestis* and PA from *B. anthracis*, in a structurally stable context. Properly folded and soluble, the triple antigen retained the functional and immunogenicity properties of all three antigens. Remarkably, two doses of this immunogen adjuvanted with Alhydrogel^®^ elicited robust antibody responses in mice, rats, and rabbits and conferred complete protection against inhalational anthrax and pneumonic plague. No significant antigenic interference was observed. Furthermore, we report, for the first time, complete protection of animals against *simultaneous* challenge with *Y. pestis* and the lethal toxin of *B. anthracis*, demonstrating that a single biodefense vaccine can protect against a bioterror attack with weaponized *B. anthracis* and/or *Y. pestis*. This bivalent anthrax–plague vaccine is, therefore, a strong candidate for stockpiling, after demonstration of its safety and immunogenicity in human clinical trials, as part of national preparedness against two of the deadliest bioterror threats.

## Introduction

*Bacillus anthracis* and *Yersinia pestis* are two Tier-1 biothreat agents that pose a great risk to public health due to their exceptionally high virulence (1–4). *B. anthracis*, a Gram-positive bacterium, is the causative agent of anthrax, and *Y. pestis*, a Gram-negative bacterium, is the etiological agent of plague. Both are deadly diseases and cause rapid death, in 3–6 days, of 85–100% of exposed individuals, unless antibiotics are administered within 20–24 h after the onset of symptoms (1–5). Intentional release of these organisms as a bioweapon could lead to massive deaths, public panic, and social chaos (1–4). The best way to offset such an attack is to vaccinate people prior to the attack. Vaccination is also essential after the attack to minimize further casualties and to mitigate additional attacks (6). Consequently, stockpiling of vaccines against anthrax and plague has been a national priority since the anthrax attacks of September 2001 (1–4).

There are currently no Food and Drug Administration-approved anthrax or plague vaccines for mass vaccination in humans. The BioThrax vaccine approved for anthrax in 1970s, anthrax vaccine alum (AVA)-adsorbed, has been used for high-risk individuals such as the military (7). This vaccine consists of a filtered crude culture supernatant of *B. anthracis* strain V770-NP1-R, but it exhibits significant reactogenicity in vaccinated individuals (7–9). A reformulated version of BioThrax vaccine (Emergent BioSolutions, Gaithersburg, MD, USA) was recently approved for humans (18–65 years of age) to prevent disease following suspected or confirmed exposure to *B. anthracis* in conjunction with recommended antibiotic treatment(s) (10). The use of this reformulated vaccine is also currently limited to military and high-risk health-care workers (10). Unfortunately, these vaccines require multiple initial doses and subsequent boosters to maintain protective immunity (7). Similarly, a killed whole cell plague vaccine was in use in the past, also for military and laboratory personnel in the United States, but was discontinued due to high reactogenicity and because its protective effect against bubonic plague did not extend to the deadlier pneumonic form of the disease (11). A live-attenuated plague vaccine, EV76, which is protective against both bubonic and pneumonic plague is used in some parts of the world where plague is endemic, but it is also associated with severe side effects (12, 13).

**Figure 1:**
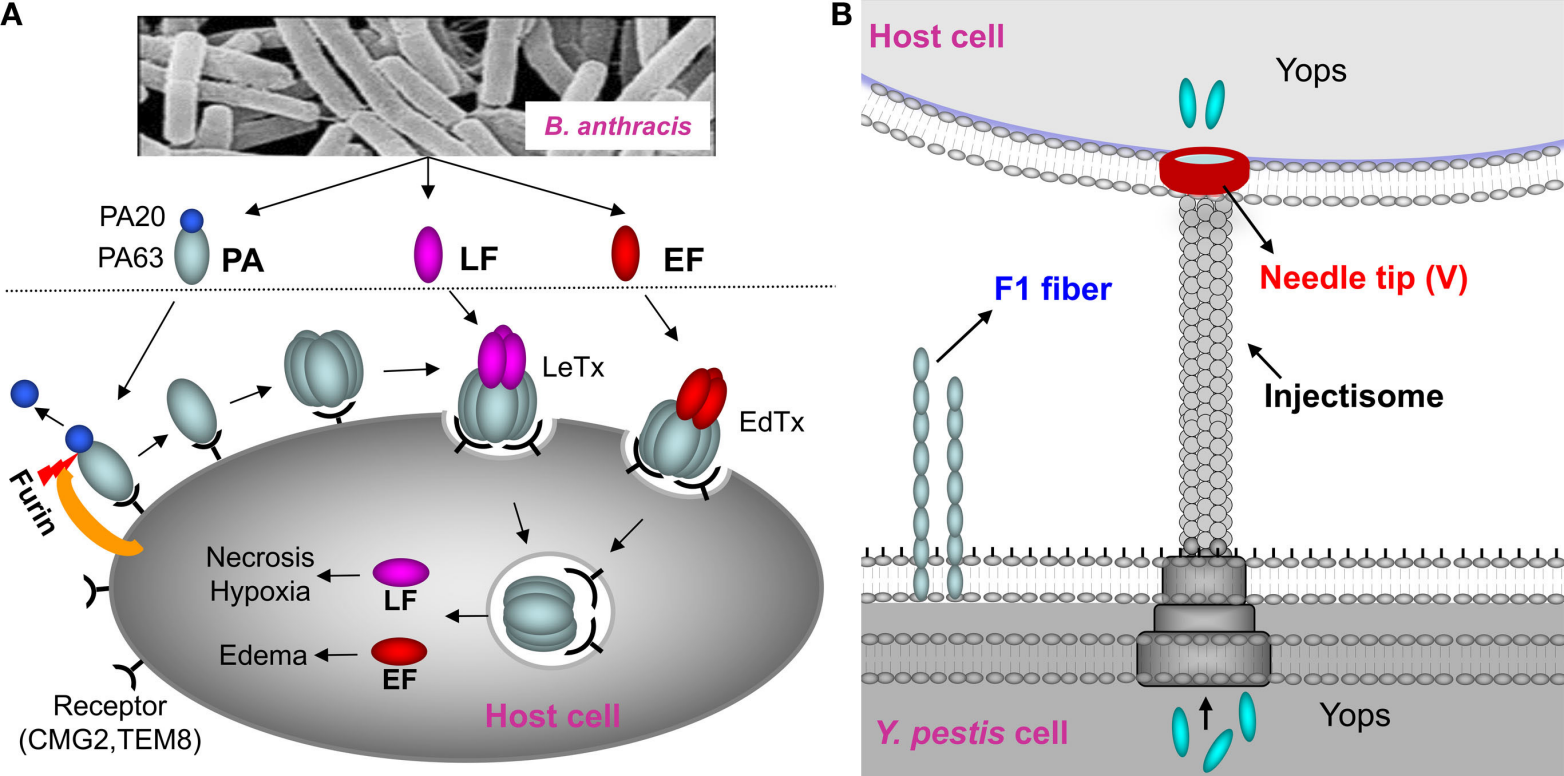
Schematic of anthrax toxin pathway and *Yersinia pestis* surface components targeted for vaccine design. **(A)** Schematic of anthrax toxin pathway. The protective antigen (PA), a key component of the lethal toxin (LeTx) of *Bacillus anthracis*, has been the principal target for the anthrax vaccines. Once bound to the host receptors CMG2 and TEM8, PA is cleaved by furin to generate PA20 (20 kDa) and PA63 (63 kDa). PA63 then oligomerizes to produce a heptamer or octamer that then interacts with lethal factor (LF) and edema factor (EF) to form the LeTx or edema toxin (EdTx), respectively. Translocation of LF and EF through the PA heptamer/octamer channel into the host cell cytosol results in toxic effects. **(B)** *Y. pestis* surface components targeted for vaccine design. F1 is the structural unit of the capsular layer. V forms a pore at the tip of the injectisome needle and facilitates translocation of *Yersinia* outer proteins (Yops) into the host cell. F1 and V are two principal targets for the plague subunit vaccines.

In recent years, the focus has been shifted to subunit vaccines containing pure recombinant proteins. The protective antigen (PA) has been the principal target for improved anthrax vaccines (8, 9). PA is the host receptor-binding component of the tripartite anthrax toxin that consists, in addition, of lethal factor (LF) and edema factor (EF) (14) (Figure 1A). Numerous studies have documented that antibodies against PA alone are sufficient to completely protect animals against lethal, aerosolized *B. anthracis* Ames spore challenge (6, 15). However, the instability of recombinant PA (rPA) when adsorbed on aluminum hydroxide gel and the variable immune responses in humans remained as a barrier for licensing an rPA anthrax vaccine (16, 17). Recombinant plague vaccines typically combine two surface-exposed antigens of *Y. pestis*, the capsular protein Caf1 (or F1; 15.6 kDa) and the low calcium response V antigen, LcrV (or V; 37.2 kDa) (11, 18) (Figure 1B). F1 assembles into fibers to form an outer capsular layer, allowing the bacterium to adhere to the host cell and escape phagocytosis (19). The V antigen forms an oligomeric “pore” at the tip of the “injectisome” needle of the *Y. pestis* type 3 secretion system through which the effector proteins (*Yersinia* outer proteins) are delivered into the host cell cytosol (20, 21) (Figure 1B). Antibodies against F1 and V provide protection against *Y. pestis* infection, although, based on literature, cellular immunity also seems to play a role in providing protective immunity (11, 18, 22). Two types of recombinant F1/V vaccines have been formulated; a mixture of F1 and V proteins or a single protein containing both F1 and V, the F1–V fusion protein (23–25). A major concern for licensing these vaccines is that the fibrous F1 protein forms heterogeneous aggregates that might compromise the quality of the vaccines and lead to variable and insufficient immune responses (24, 26, 27).

Another major problem in developing these biodefense vaccines is the need for two separate vaccines requiring two completely different manufacturing processes. For national preparedness against potential bioterror threats, it would be highly desirable to design a single multivalent vaccine that can provide protection against both the pathogens, *B. anthracis* and *Y. pestis*. Such a vaccine would require a single manufacturing process, fewer immunizations, and would be cost-effective. It would also greatly reduce time and effort in expensive human clinical trials and the downstream licensing and other regulatory processes. Furthermore, and perhaps most significant, it would streamline the systems for stockpiling, field delivery, and mass vaccination of humans.

Here, we report a new approach to design a single biodefense vaccine against inhalation anthrax and pneumonic plague. Using structure-based immunogen design, we engineered a triple antigen containing mutated F1 (26), V (21), and PA (28) that folded into a soluble protein and retained full functionality. The triple antigen generated robust antigen-specific immune responses and provided complete protection against anthrax and plague in three different animal models. Furthermore, by using a dual challenge model in which the animals were simultaneously administered with lethal doses of both anthrax lethal toxin (LeTx) and *Y. pestis* CO92, we demonstrate that our vaccine provided complete protection against both anthrax and plague. Our studies provide the first proof-of-concept data that a bivalent anthrax–plague vaccine can potentially protect vaccinees in the event of a bioterror attack with weaponized *B. anthracis* and/or *Y. pestis*. This bivalent vaccine, therefore, is a strong candidate for stockpiling as part of our national preparedness against bioterrorism threats.

## Results and Discussion

### Construction of an Anthrax–Plague Triple Antigen

**Figure 2:**
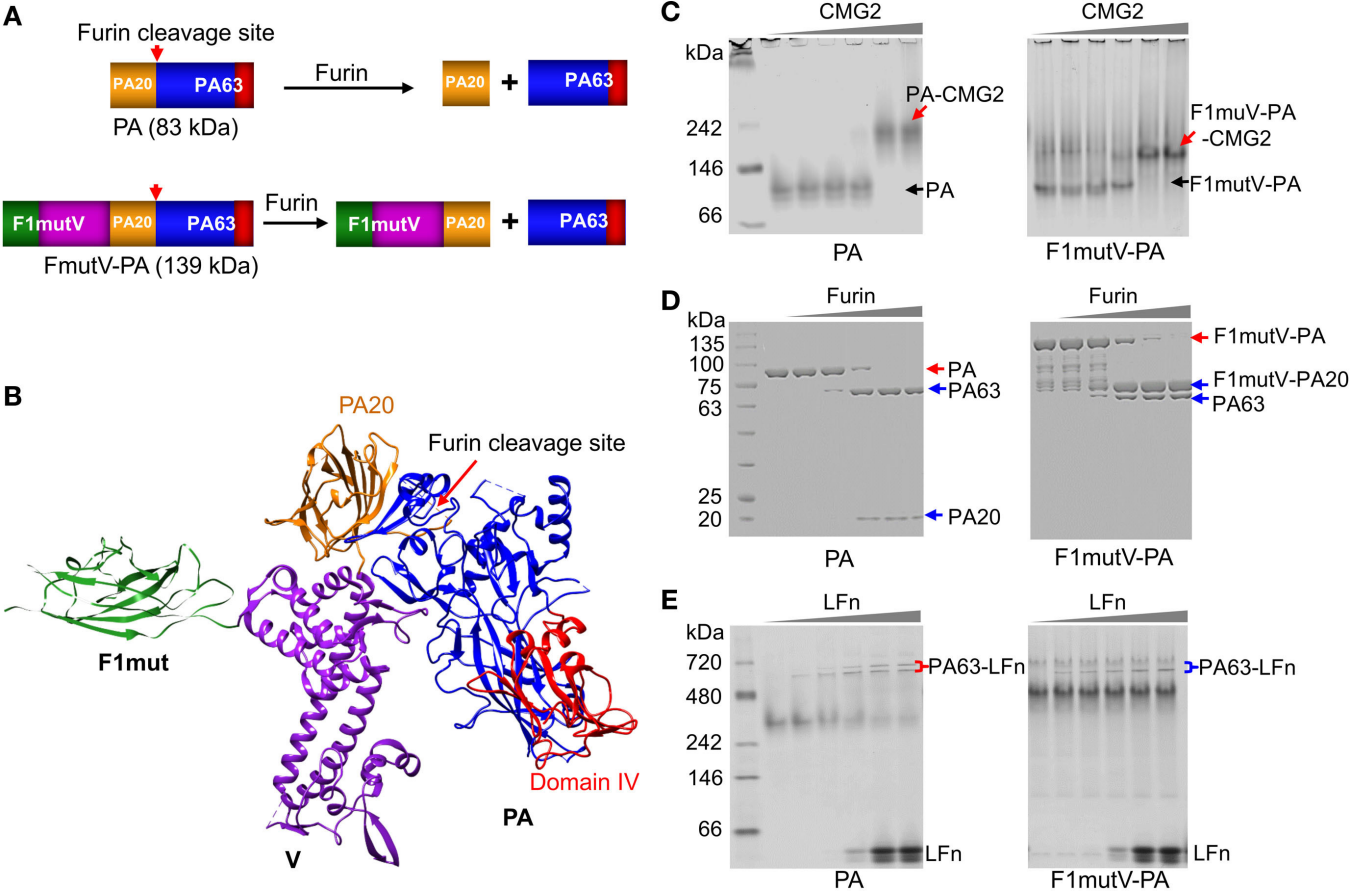
Construction and characterization of an anthrax–plague triple antigen. **(A)** Schematic of protective antigen (PA) and F1mutV-PA recombinant constructs. The PA20 domain of PA is shown in yellow, the PA63 domain is shown in blue, PA domain IV is shown in red, F1mut is shown in green, and V is shown in purple. Furin cleavage site and its cleavage products are also indicated. **(B)** Structural model of F1mutV-PA triple antigen. The model was manually generated using Chimera with structures of F1 (PDB ID: 1Z9S), V (PDB ID: 4JBU), and PA (PDB ID: 1ACC). **(C)** Binding to PA receptor, CMG2. The purified PA or F1mutV-PA proteins were incubated with increasing amounts of CMG2 and interactions between the PA proteins and CMG2 were analyzed by native-PAGE. PA-CMG2 and F1mutV-PA-CMG2 complexes are marked with red arrows. PA and F1mutV-PA are marked with black arrows. **(D)** Furin cleavage. The PA or F1mutV-PA proteins were treated with increasing amounts of furin and the cleavage products were analyzed by SDS-PAGE. Positions of the PA and F1mutV-PA bands are marked with red arrows and the positions of the cleaved products PA63, PA20, and F1mutV-PA20 bands are marked with blue arrows. **(E)** Binding to N-terminal domain of lethal factor (LFn). The PA and F1mutV-PA proteins were first treated with furin to release PA63 and then incubated with increasing amounts of LFn. Interactions between the PA63 heptamer/octamer and LFn were analyzed by native-PAGE. The PA63–LFn complexes are marked with braces. The SDS-PAGE and native gels were stained with Coomassie blue R-250 and Coomassie blue G-250, respectively.

To create a bivalent anthrax–plague vaccine, we fused in-frame the coding sequences corresponding to F1mut, V, and PA (Figure 2A; Figure S1A in Supplementary Material). The F1mut was previously designed by deleting the N-terminal β-strand residues 1–14 of F1 and fusing them to the C-terminus with a Ser-Ala linker in between. Consequently, the β-strand is reoriented such that it fits into its own β-sheet cleft (intramolecular complementation) rather than that of the adjacent F1 subunit. In addition, residues 15–21 were duplicated at the C-terminal end to restore any potential T-cell epitope that might have been compromised during the β-strand switch. As a result, F1mut folds into a monomer instead of polymerizing as a linear fiber and retains full immunogenicity (26, 29). The V and PA sequences of the triple antigen correspond to native full-length sequences (21, 28).

Our goal was to retain the structural and functional integrity of all three antigens so that their immunogenicity and protective efficacy were not compromised. To achieve this, the C-terminus of F1mutV (56 kDa) was fused to the N-terminus of PA (83 kDa) with a flexible Glu-Ala-Ser-Ala linker in the middle (see Figure S1 in Supplementary Material and the Section “Materials and Methods” for additional details). Based on structural and bioinformatics analyses, we predicted that this orientation would be optimal because the C-terminal PA domain IV, which recognizes the host receptors CMG2 (capillary morphogenesis gene-2) and TEM8 (tumor endothelial marker-8), will encounter minimal, if any, steric hindrance (Figure 2B) (30, 31). Recognition of these receptors is the first step in the anthrax toxin intoxication pathway within the host cell and essential for furin cleavage of the N-terminal domain of PA to generate PA20 (20 kDa) and PA63 (63 kDa) (Figures 1A,B) (32). PA63 oligomerizes to produce heptamers and octamers that then interact with LF and EF (Figure 1A) (14). Although in our construct the F1mutV protein is attached to the N-terminus of PA (Figure 2A), we reasoned, based on the linear domain arrangement of F1 and V proteins as determined by the X-ray structures (21, 33), that the furin cleavage site at PA residues RKKR [amino acids (aa) 164–167] should remain accessible to the protease (Figure 2B). The F1mutV-PA protein was expressed in *Escherichia coli* BL21-codon plus (DE3)-RIPL cells and purified from the soluble fraction at the yield of 5–10 mg/L. Remarkably, the 139 kDa F1mutV-PA protein consisting of seven domains belonging to three different proteins (Figure 2B) was soluble and existed mainly as a monomer in solution as determined from the elution profile following size-exclusion chromatography (Figure S1B in Supplementary Material).

A series of quantitative biochemical analyses were performed to verify the functionality of F1mutV-PA. First, F1mutV-PA bound to the soluble external domain of CMG2 equivalently as the rPA at different ratios of F1mutV-PA:CMG2, generating a high-molecular weight complex (Figure 2C, red arrows). Second, F1mut-PA and rPA had similar sensitivity to various concentrations of furin (Figure 2D). Whereas rPA was cleaved to PA63 and PA20, F1mutV-PA was cleaved to 76 kDa F1mutV-PA20 and PA63. Third, as in the case of the rPA, the PA63 generated by cleavage of F1mutV-PA bound to LFn (N-terminal PA-binding domain of LF), resulting in the formation of PA63–LFn complexes (Figure 2E). Collectively, these results demonstrated that the biochemical properties of the fusion protein F1mutV-PA remained similar to rPA.

### The F1mutV-PA Triple Antigen Is Highly Immunogenic in Mice

**Figure 3:**
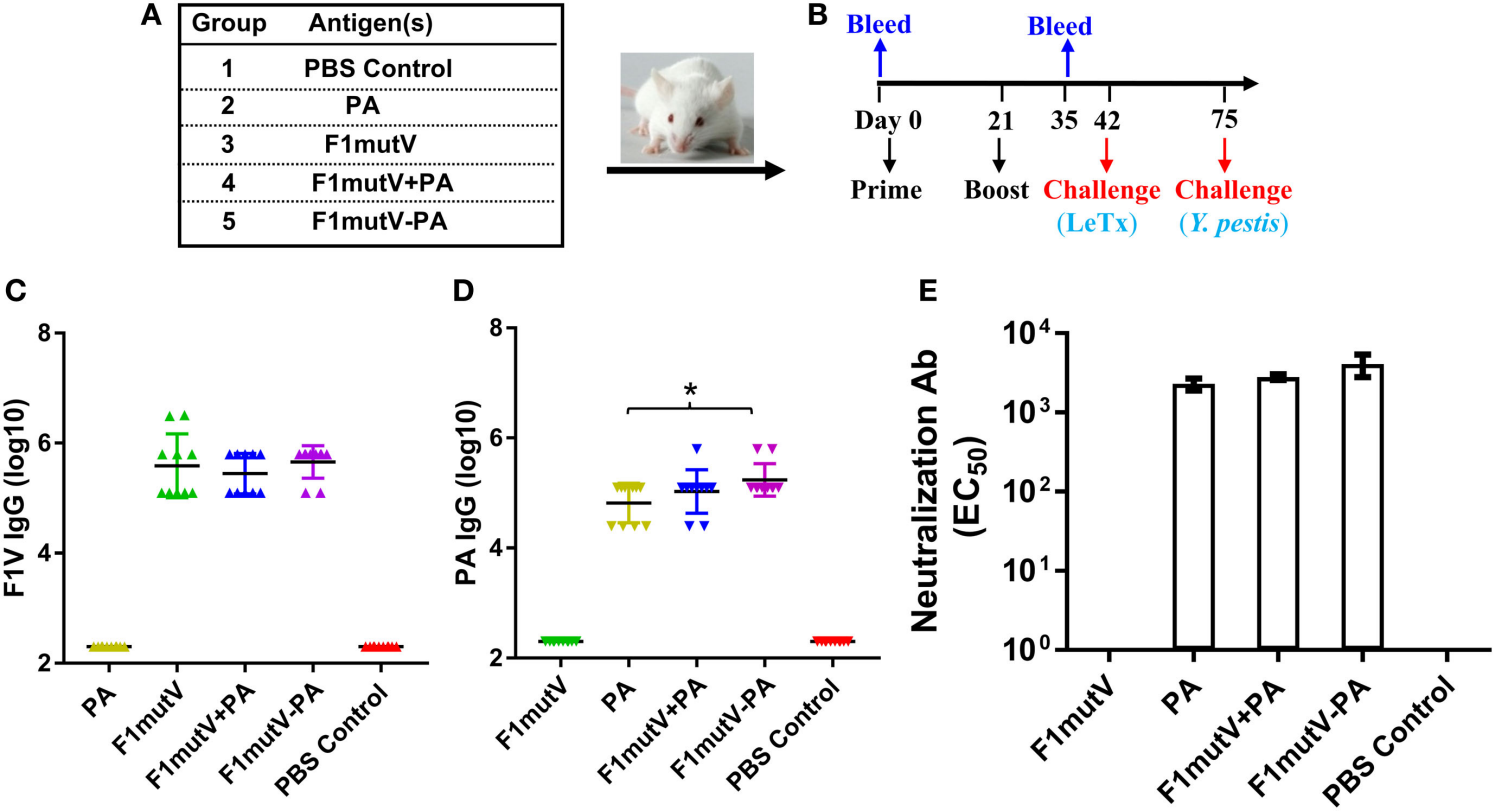
The F1mutV-protective antigen (PA) triple antigen is highly immunogenic in mice. **(A)** Vaccine formulations used in various immunized mouse groups. The protein combinations used for each group are shown. **(B)** The immunization scheme. Mice (*n* = 10) were immunized (intramuscular) on days 0 and 21. Sera were collected on days 0 and 35 for antibody analysis. Animals were challenged with lethal toxin (LeTx) on day 42 followed by *Yersinia pestis* CO92 on day 75. **(C)** F1V-specific antibody titers. **(D)** PA-specific antibody titers. **(E)** LeTx-neutralizing antibody titers. Error bars represent SD. “*” denotes *p* < 0.05 (analysis of variance).

Balb/c mice (*n* = 10/group) were immunized by the intramuscular (i.m.) route with 50 µg of F1mutV-PA and were boosted once on day 21. Mice immunized with PA (25 µg) alone, F1mutV (25 µg) alone, or a mixture of F1mutV and PA (F1mutV + PA, 25 µg of each) served as control groups (Figures 3A,B; see Section “Materials and Methods” for details). The latter group allowed assessment of our bivalent vaccine formulations relative to a simple mixture of the two antigens.

All of the F1mutV immunogens elicited high and comparable levels of F1mutV-specific IgG antibodies, up to an end point titer of ~3 × 10^6^ (Figure 3C). The PA antigens similarly elicited high antibody titers. However, significantly, the triple antigen F1mutV-PA generated higher PA-specific antibody titers when compared to the PA group (Figure 3D, *p* < 0.05). The naïve animals, as expected, showed no antibodies to either PA or F1mutV (Figures 3C,D). Similarly, the animals immunized with PA alone had no F1mutV-specific antibodies and *vice-a-versa* (Figures 3C,D).

A LeTx neutralization assay (TNA) was performed to determine LeTx-neutralizing activity by anti-PA antibodies present in the sera of the immunized mice. Previous studies demonstrated that the levels of LeTx-neutralizing antibodies correlated with protection against inhalational *B. anthracis* challenge (34). All of the groups immunized with the PA antigen demonstrated strong LeTx neutralization titers (Figure 3E). The naïve animals (PBS group) or the F1mutV-immunized animals, as expected, were negative for toxin neutralization.

**Figure 4:**
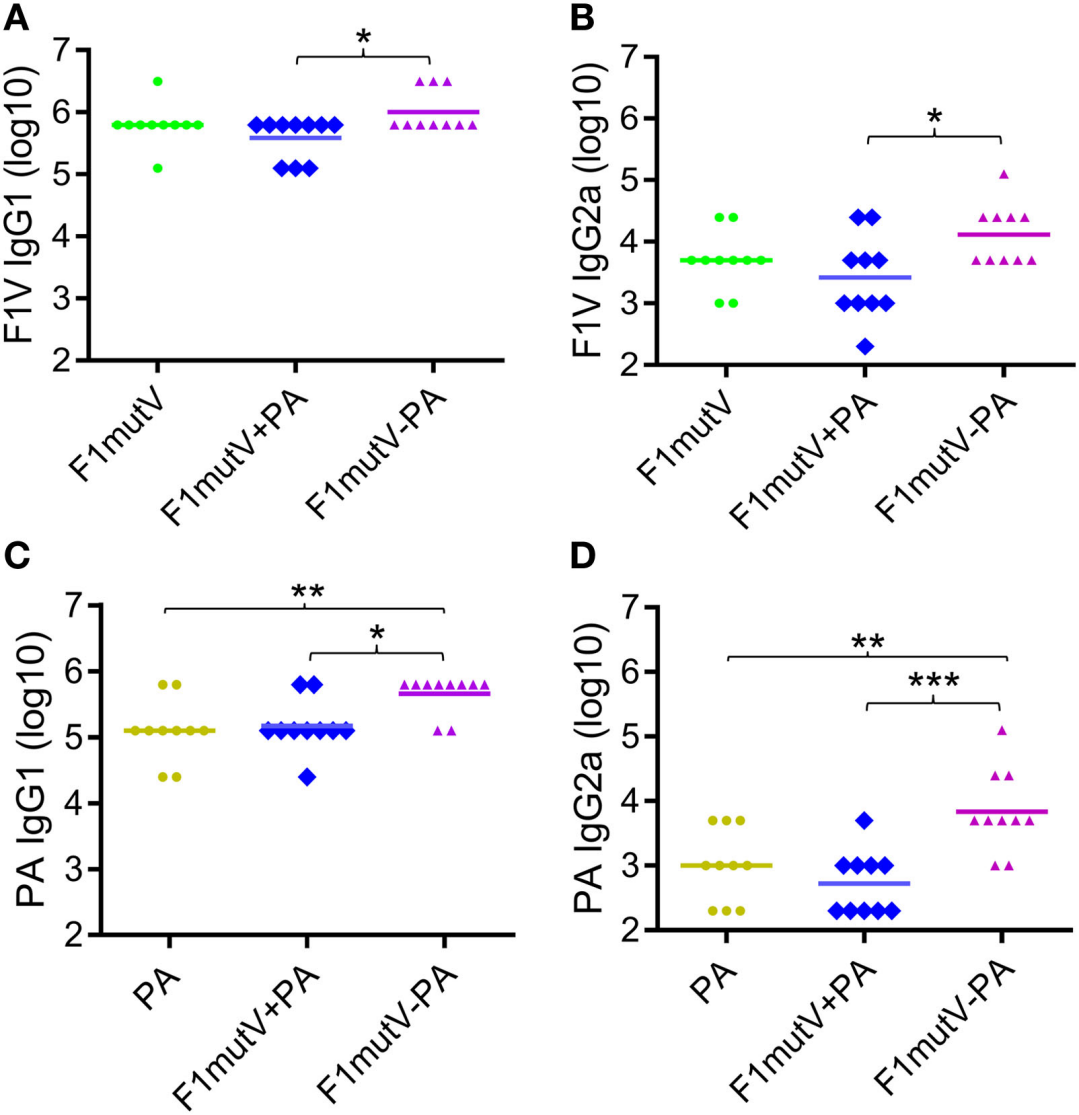
Subtype specificity of antibodies elicited by F1mutV-protective antigen (PA) triple antigen in mice. The panels show F1V-specific IgG1 (T_H_1) **(A)** and IgG2a (T_H_2) **(B)** titers and PA-specific IgG1 **(C)** and IgG2a **(D)** titers. Mice were immunized (intramuscular) according to Figures 3A,B. Sera were collected according to Figure 3B and analyzed by ELISA. “*”, “**”, and “***” denote *p* < 0.05, *p* < 0.01, and *p* < 0.001, respectively (analysis of variance).

We measured IgG antibody subtypes (IgG1 and IgG2) that represent stimulation of T_H_2 and T_H_1 immune responses, respectively. Both might be important for protection against *Y. pestis* infection (35, 36), and probably also against *B. anthracis* infection (37). With this in mind, we determined the IgG subclass of the induced antibodies by ELISA (Figure 4). In mice, IgG2a titer represents T_H_1 response whereas IgG1 reflects the T_H_2 response. Our data showed that the F1mutV-PA group elicited higher levels of IgG1 and IgG2a antibody titers when compared to the F1mutV + PA group (Figures 4A,B; *p* < 0.05). Similar pattern was observed with respect to the PA-specific IgG1 and IgG2a (Figure 4C, *p* < 0.05; Figure 4D, *p* < 0.001). No significant differences of immunogen specific antibodies between the control F1mutV + PA vs F1mutV or PA groups were observed (Figure 4).

These results indicated that the soluble F1mutV-PA triple antigen showed an overall bias toward T_H_2 responses, as has been generally observed with many subunit vaccines (38). Notably, however, F1mutV-PA elicited significantly greater IgG2a against both F1V and PA antigens, when compared to the F1mutV + PA group or the PA group (Figures 4B,D). Thus, the triple antigen F1mutV-PA is a more potent immunogen when compared to the individual antigens or a simple mixture.

### The Bivalent Vaccine Protects Mice against Challenges with LeTx and *Y. pestis* CO92

Since our goal was to assess protection against both inhalation anthrax and pneumonic plague, it became imperative to establish appropriate challenge models. In previous reports on dual anthrax–plague vaccines, groups of animals were immunized with mixtures of PA, F1, and V (39–42) but challenged separately with either *B. anthracis* [intratracheal (39) or subcutaneous (40) administration of spores prepared from the non-encapsulated toxigenic Sterne strain] or *Y. pestis* [intraperitoneal (39) or subcutaneous (40–42) injection]. However, this model would not provide an accurate assessment of dual protection because the animals were not exposed to both the agents. Therefore, we developed two new challenge models using mice and rats; a sequential dual challenge model in which the animals were first exposed to one agent and the survivors were then exposed to the second agent, and a simultaneous dual challenge model in which the animals were exposed to both the threat agents at the same time (see Table S1 in Supplementary Material for the details regarding dose, route, and schedule). We chose Balb/c mice and Brown Norway rats because both these animal strains are highly susceptible to LeTx and *Y. pestis* bacterial challenge and the protection outcomes provide good benchmarks for evaluation of vaccine efficacy (43–45). Since the most virulent form of *Y. pestis* is the aerosolized form (3), intranasal (i.n.) challenge was used to evaluate vaccine efficacy.

**Figure 5:**
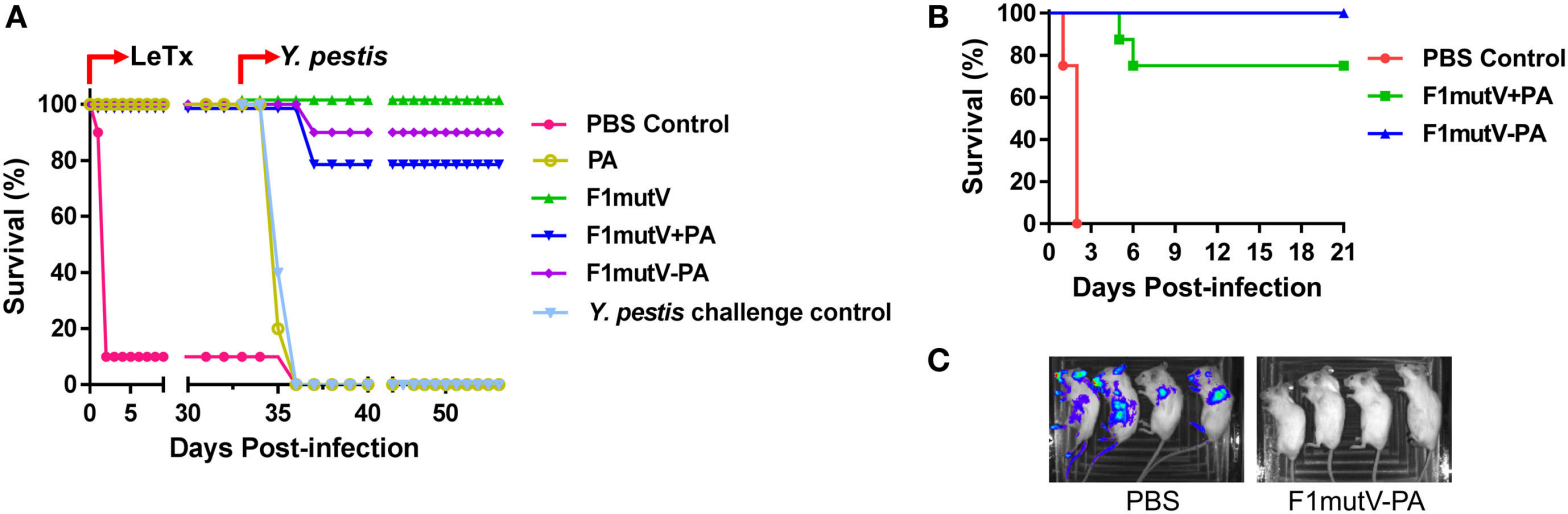
The bivalent anthrax–plague vaccine protects mice against challenges with lethal toxin (LeTx) and *Yersinia pestis* CO92. **(A)** Survival of mice against anthrax LeTx and plague sequential challenge. Mice (*n* = 10/group) were immunized (intramuscular, i.m.) according to Figure 3B and challenged with 1 LD_100_ LeTx (intraperitoneally) on day 42 postimmunization, followed by intranasal (i.n.) challenge with 400 LD_50_ *Y. pestis* CO92 on day 75 postimmunization. **(B)** Survival of mice against simultaneous anthrax LeTx and plague challenge. Mice (*n* = 8/group) were immunized (i.m.) with F1mutV-protective antigen (PA) or F1mutV-PA. On day 44 postimmunization, mice were simultaneously challenged with 1 LD_100_ LeTx (i.p.) and 200 LD_50_ *Y. pestis* (i.n.). **(C)** *In vivo* imaging of challenged mice. Luciferase expression by *Y. pestis* in representative mice from naïve control (PBS) and the F1mutV-PA-immunized groups on day 3 postchallenge is shown. The PBS control group used for imaging here was challenged with *Y. pestis* alone to minimize any interference from LeTx. Note that death of animals challenged with *Y. pestis* alone occurred in 4 days, whereas it occurred in 2 days when LeTx was included in the challenge as the toxin leads to early animal lethality **(B)**.

For sequential challenge, mice were immunized as per the above scheme (Figure 3B) and injected intraperitoneally (i.p.) with 1 LD_100_ of LeTx (1:1 mixture of PA and LF, 100 µg each) 2 weeks after the boost. The F1mutV–PA and other PA-immunized groups were 100% protected against LeTx challenge whereas 90% of the naïve group mice died within 2 days of toxin challenge (Figure 5A). Thirty-three days later, the animals were challenged with the second pathogen, ~400 LD_50_ [1 LD_50_ = 100 colony-forming units (CFU) in Balb/c mice] of *Y. pestis* CO92, a highly lethal strain, by i.n. administration. The naïve mice and the F1mutV-immunized mice were used as negative and positive controls, respectively. The LeTx-challenged PA group provided another (negative) control. The F1mutV-PA group showed 90% protection (one of ten mice died) whereas the F1mutV + PA group showed 80% protection (two of ten mice died). The naïve and PA-immunized animals showed 100% death within 4 days post-*Y. pestis* CO92 challenge (Figure 5A). The one survivor in the LeTx-challenged PBS group died 3 days post *Y. pestis* challenge (Figure 5A). As reported previously (26), all the control F1mutV-immunized mice were fully protected.

To test the protective ability of the vaccines in a dual challenge model involving simultaneous exposure, we challenged mice with LeTx and *Y. pestis* CO92 at the same time. Mice (*n* = 8) were immunized twice with antigens or PBS as per the same scheme (Figure 3B) and challenged with both LeTx (1 LD_100_, i.p. administration) and *Y. pestis* CO92 (200 LD_50_, i.n. administration) 23 days after the boost (day 44 postimmunization). In addition, a second PBS group was used as a control for challenge with *Y. pestis* (200 LD_50_, i.n.) alone. As shown in Figure 5B, the PBS control mice challenged with both LeTx and *Y. pestis* died within 2 days post challenge. But the PBS control mice challenged with *Y. pestis* alone died by 4 days. The bivalent F1mutV-PA vaccine provided 100% protection (eight out of eight mice), while the F1mutV + PA mixture provided 75% (six out of eight mice) protection (Figure 5B). Furthermore, the survivors showed the clearing of *Y. pestis* bacteria by 3 days postchallenge (Figure 5C). The *Y. pestis* CO92 strain used in the challenge experiment contained a luciferase expression cassette for imaging the bacteria *in vivo* in real time (46). The immunized animals were negative for bioluminescence, whereas the PBS control mice, which were challenged with *Y. pestis* alone, showed bacterial dissemination throughout the body (Figure 5C; see legend to Figure 5C for more details).

The above data sets demonstrated that the bivalent anthrax–plague vaccine was highly immunogenic in the mouse model and conferred complete protection upon simultaneous double challenge with LeTx and *Y. pestis* CO92.

### The Bivalent Anthrax–Plague Vaccine Provides Complete Protection against both LeTx and *Y. pestis* CO92 in Brown Norway Rats

**Figure 6:**
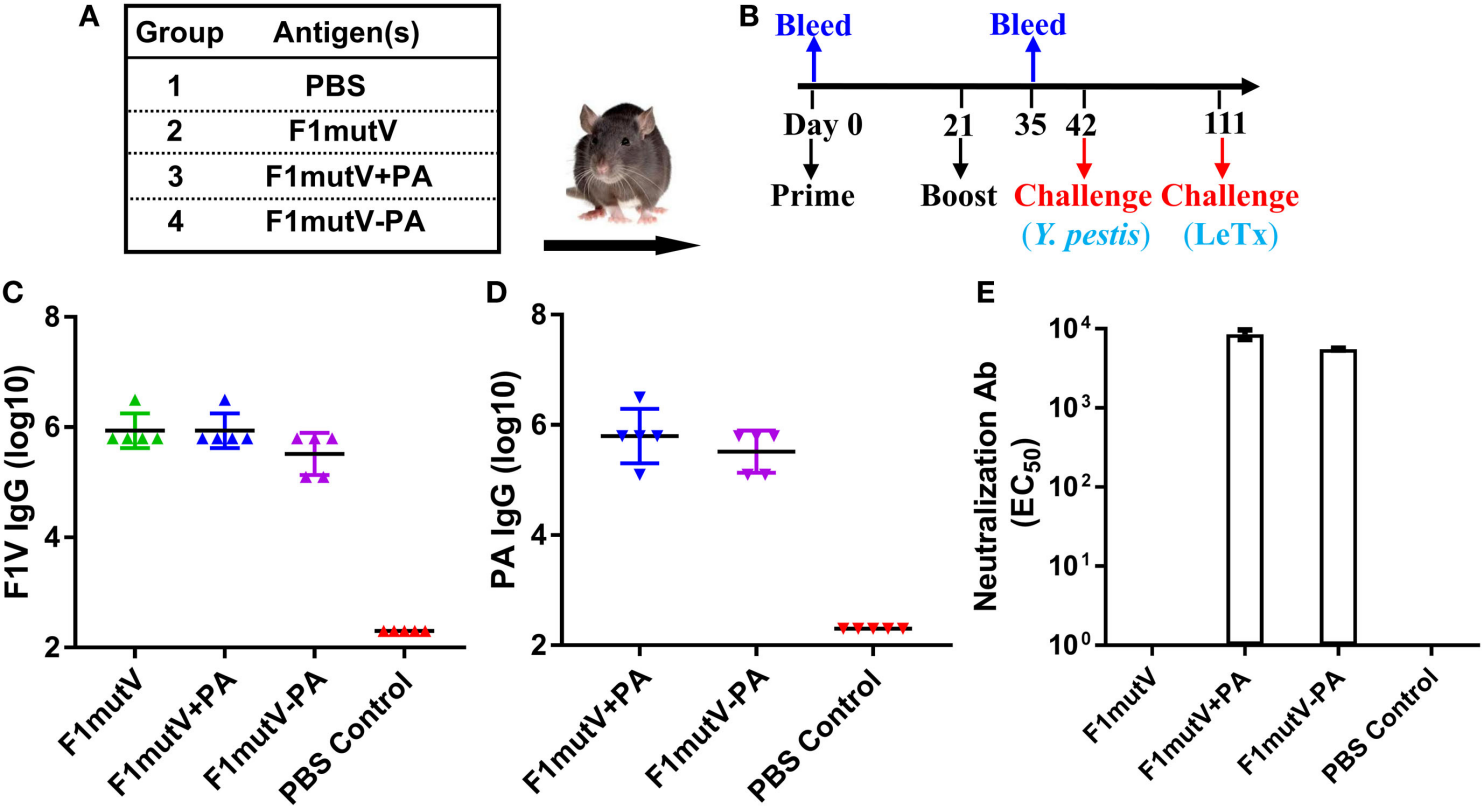
The F1mutV-protective antigen (PA) triple antigen is highly immunogenic in rats. **(A)** Vaccine formulations used in various immunized Brown Norway rat groups. The protein combinations used for each group are shown. **(B)** The immunization scheme. Rats (*n* = 9) were immunized (intramuscular) on days 0 and 21. Sera were collected on days 0 and 35 for antibody analysis. Animals were challenged with *Yersinia pestis* (intranasal) on day 42 followed by lethal toxin (LeTx) (intravenous) on day 111. **(C)** F1V-specific antibody titers. **(D)** PA-specific antibody titers. **(E)** LeTx-neutralizing antibody titers. Error bars represent SD.

Rat, the natural host of *Y. pestis* through infection by rat fleas, is one of the most reliable models to assess the protective efficacy of vaccines against plague (43). To further evaluate our bivalent vaccine, Brown Norway rats (*n* = 9) were immunized and challenged using the scheme shown in Figures 6A,B. As in mice, the immunogens induced high levels of total antigen-specific IgG titers, up to ~3 × 10^6^ (Figures 6C,D). The level of anti-F1V IgG was comparable among all the groups and no significant differences were observed (Figure 6C). Similarly, no significant difference in anti-PA IgG was observed among immunized groups (Figure 6D). Consistent with the latter, all the PA groups generated high and comparable LeTx-neutralizing antibodies (EC_50_ of 4,300–8,500) (Figure 6E). The naïve animals, as expected, were negative for the antigen-specific or LeTx-neutralizing antibodies. Similarly, the PA-alone animals were negative for F1mutV antibodies and the F1mutV-alone animals were negative for PA and LeTx-neutralizing antibodies.

**Figure 7:**
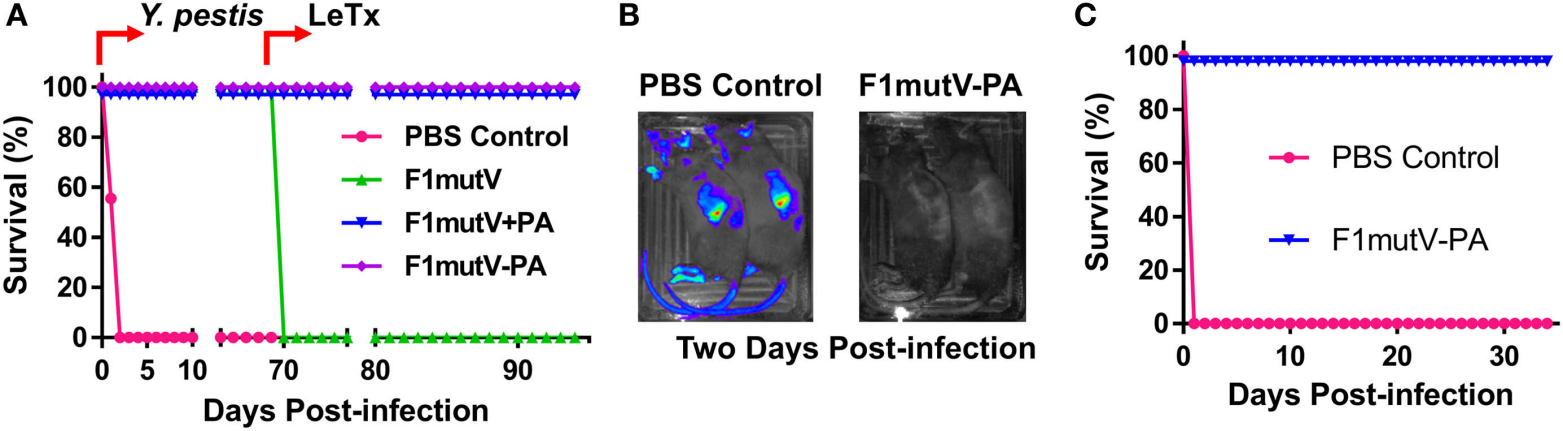
The bivalent anthrax–plague vaccine provides complete protection against both lethal toxin (LeTx) and *Yersinia pestis* CO92 in Brown Norway rats. **(A)** Survival of rats against anthrax LeTx and plague sequential challenge. Rats (*n* = 9) were challenged (intranasal, i.n.) with 400 LD_50_ *Y. pestis* CO92, followed by intravenous (i.v.) injection of 1 LD_100_ LeTx. **(B)** *In vivo* imaging of infected animals. Luciferase expression in representative rats from the naïve control (PBS) and the F1mutV-protective antigen (PA) immunized groups 2 days after *Y. pestis* CO92 challenge is shown. **(C)** Survival of rats against simultaneous anthrax LeTx and plague challenges 3 weeks after the boost. Rats (*n* = 6) were immunized according to Figure 6B and challenged simultaneously with 1 LD_100_ (i.v.) of LeTx and 400 LD_50_ *Y. pestis* CO92 (i.n.).

The protective efficacy of the bivalent anthrax–plague vaccine in Brown Norway rats was first tested by the sequential dual challenge model (Figure 7A; Table S1 in Supplementary Material). The animals were subjected to i.n. challenge with 400 LD_50_ of *Y. pestis* CO92. F1mutV-PA and F1mutV + PA showed 100% protection as was the F1mutV-immunized group used as a positive control, whereas all the rats in the naïve group died within 2 days postchallenge. The clearance of *Y. pestis* CO92 from the rats was also monitored through live imaging of the *in vivo*-expressed luciferase (Figure 7B). The data showed that 2 days postchallenge with *Y. pestis*, all immunized rats cleared *Y. pestis* CO92 as indicated by the lack of a detectable luciferase signal, while all control rats had strong luciferase signals throughout the body. The survived rats were then further challenged with 1 LD_100_ of LeTx (7.5 µg each of PA and LF) by intravenous (i.v.) injection on day 70 post*-Y. pestis* CO92 challenge. All the rats immunized with the bivalent vaccine or the mixture survived (Figure 7A), but rats in the F1mutV group (negative control) died within 2 h of the LeTx challenge.

The protection efficiency of F1mutV-PA against *B. anthracis* and *Y. pestis* was further determined by simultaneously challenging with both LeTx (1 LD_100_, i.v.) and *Y. pestis* CO92 (400 LD_50_, i.n.) in an independent experiment (*n* = 6). The F1mutV-PA bivalent vaccine showed 100% protection (Figure 7C; Table S1 in Supplementary Material). Furthermore, at the end of the study, various organs (lungs, liver, and the spleen) were examined for the presence of *Y. pestis* by plate count, and no viable bacteria were detected.

The above sets of data demonstrated that our bivalent F1mutV-PA anthrax–plague vaccine is highly immunogenic in the Brown Norway rat, the natural host of *Y. pestis*, and the PA- and F1mutV-specific antibodies elicited provided complete protection against sequential or simultaneous LeTx and *Y. pestis* CO92 challenges.

### The Triple Antigen Vaccine Provides Complete Protection in the New Zealand White Rabbit Model of Inhalation Anthrax

Rodents are very sensitive to infection by *B. anthracis* bacteria that produce polyglutamic acid capsule. They succumb to encapsulated *B. anthracis* infection even if these bacteria do not produce anthrax toxin (44). Hence, lethal dose toxin challenge models are preferred for testing the efficacy of anthrax vaccines in rodents. Rabbits are a better model to determine protective efficacy of anthrax vaccines against encapsulated toxigenic *B. anthracis* as inhalation anthrax in these animals shows remarkably similar pathology to that observed in humans (47, 48). Hence, the efficacy of our bivalent anthrax–plague vaccine was tested in the New Zealand White rabbit model of inhalation anthrax (47–49).

**Figure 8:**
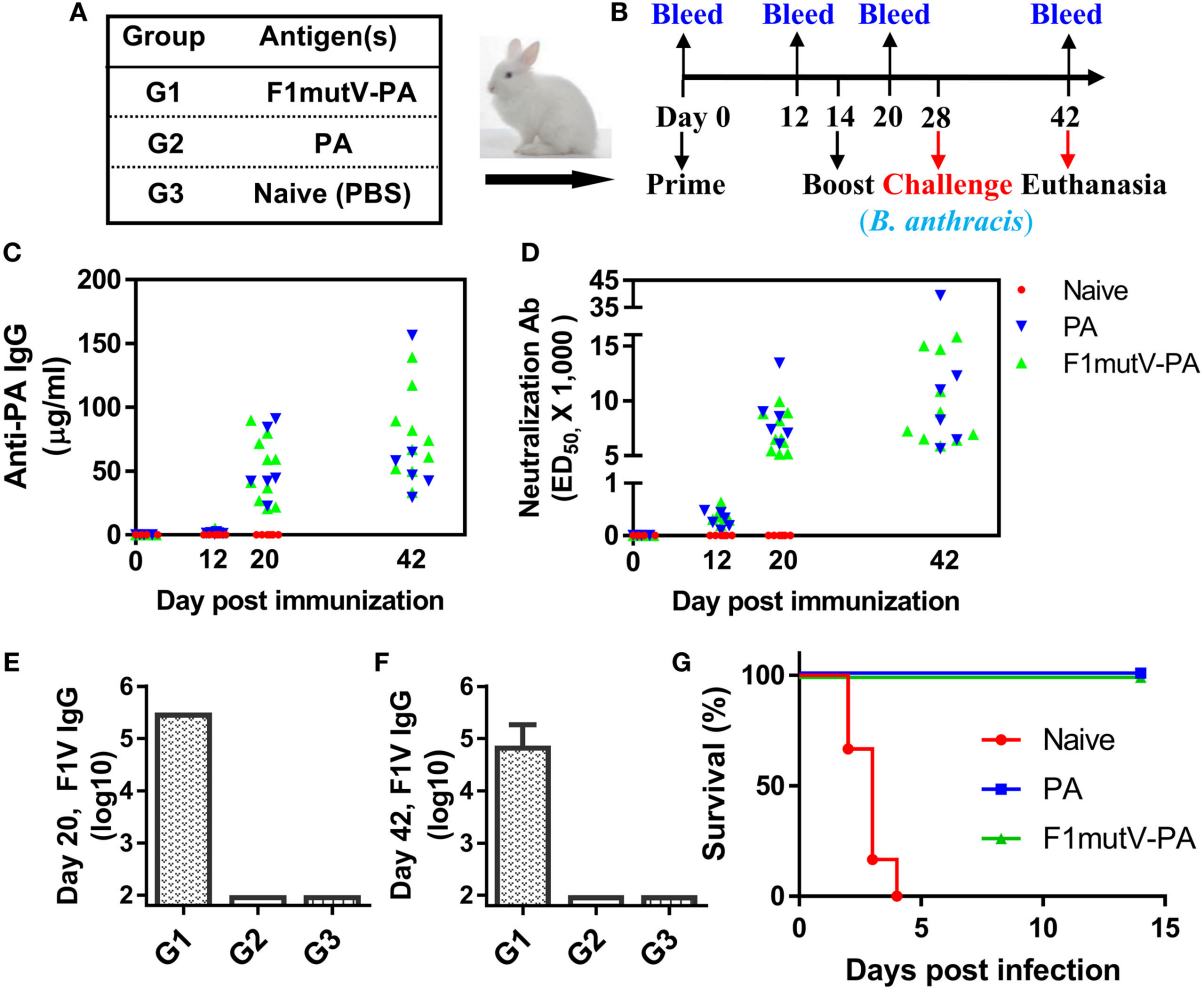
The triple antigen vaccine provides complete protection in the New Zealand White rabbit model of inhalation anthrax. **(A)** Vaccine formulations used in various New Zealand White rabbit groups. The protein combinations used for each group are shown. **(B)** Immunization scheme for rabbit study. Rabbits (*n* = 10 for group 1, and *n* = 6 for groups 2 and 3, equal numbers of males and females) were immunized on day 0 and given a boost on day 14. Animals were challenged with 200 LD_50_ of aerosolized *Bacillus anthracis* Ames spores 2 weeks after the boost. **(C)** Protective antigen (PA)-specific antibody titers. Titers for bleeds on days 0, 12, 20, and 42 are shown. **(D)** Lethal toxin-neutralizing antibody titers. **(E,F)** F1V-specific antibody titers. Titers are shown for day 20 **(E)** and day 42 **(F)**. **(G)** Survival of the rabbits challenged with 200 LD_50_ of aerosolized *B. anthracis* Ames spores. Error bars represent SD of the mean.

New Zealand White rabbits (*n* = 10 for groups 1, and *n* = 6 for groups 2 and 3, equal numbers of males and females) were primed on day 0 and boosted on day 14 by i.m. injections of F1mutV-PA (Figures 8A,B). PA was used as a positive control, while PBS served as a negative control. Sera were collected on days 0, 12, 20, and 42 (Figure 8B) and subjected to immunological analyses. The data showed that both the F1mutV-PA and PA vaccines induced high levels of anti-PA antibodies as well as LeTx-neutralizing antibodies at day 20 (Figures 8C,D). These titers are similar to that reported for the licensed AVA (50, 51).

The rabbits also induced high levels of anti-F1mutV antibodies (Figures 8E,F). At day 20, 6 days after the boost, the end point titers were in the range of 3.1 × 10^5^ (Figure 8E). There is no significant decrease in antibodies by the end of the experiment, day 42 (Figure 8F).

Rabbits were challenged two weeks after the boost with 200 LD_50_ of aerosolized *B. anthracis* Ames spores (Table S1 in Supplementary Material). All the naïve control rabbits succumbed to the anthrax disease 2–4 days postinfection, while all the vaccine immunized rabbits were completely protected (Figure 8G). Between the challenge day and the end of the study (days 28–42), vaccinated animals from Groups 1 and 2 continued to show an increase in the body weight, while control animals showed weight loss as well as body temperature changes before death (Figure S2 in Supplementary Material).

Blood samples for bacteremia were drawn before the challenge on day 27 (baseline) and on days 29–33 (1–5 days postexposure) and day 42. Vaccinated animals (Group 1 and 2) never developed bacteremia, whereas all unvaccinated control animals (Group 3) became positive for bacteremia before they succumbed to the disease (Table S2 in Supplementary Material). To determine the bacterial load of internal organs, postmortem collection of specimens was performed after scheduled euthanasia of surviving animals on study day 42 (Group 1 and 2) or after animals died due to the anthrax exposure (Group 3). All vaccinated animals from Groups 1 and 2 had cleared the agent from the lungs and did not have any bacteria in the brain, liver, or spleen (Table S3 in Supplementary Material). In contrast, tissue samples collected from unvaccinated control animals (Group 3) had very high bacterial titers indicative of systemic anthrax infection (Table S3 in Supplementary Material). In increasing order, the brain titer average was 5 × 10^6^ CFU/g, the liver average was 3 × 10^7^ CFU/g, and the highest average titer of 5 × 10^8^ CFU/g was obtained for lung and spleen samples (Table S3 in Supplementary Material). Gross necropsy and histological analyses were consistent with these data.

The above sets of data demonstrated that both our F1mutV-PA dual anthrax–plague vaccines provided 100% protection in rabbits against aerosolized *B. anthracis* Ames spore challenge.

## Conclusion

Since the deadly anthrax attacks of 2001, stockpiling of recombinant plague and anthrax vaccines has been a national priority. However, no candidate vaccines have yet been able to meet the licensing requirements. A single bivalent vaccine, rather than two different vaccines, which can protect against both Tier-1 bioterror pathogens, *B. anthracis* and *Y. pestis*, would greatly accelerate this effort. We report here such vaccine, a F1mutV-PA triple antigen, which incorporates all three key antigens, F1 and V from *Y. pestis* and PA from *B. anthracis*.

Informed by structural analyses, we engineered this immunogen in such a way that the 139 kDa protein is soluble and folded correctly to retain the biochemical functions and immunogenicity of all three antigens. The seven domain structure of the protein showed nearly the same level of activity as the native PA with respect to interaction with the host receptor CMG2, cleavage by furin protease, and binding to LFn. In addition, the immunogen elicited robust and protective immune responses in three different animal models, namely mouse, rat, and rabbit.

There have been several previous studies on developing a dual anthrax–plague vaccine, all involving a simple mixture of F1, V, and PA proteins (39–41). Both synergy and interference in antibody production have been reported when the antigens were mixed. However, none of these candidate vaccines were tested for efficacy against both the biothreat agents (39–41). We found no evidence of antigen interference with our bivalent anthrax–plague vaccine, although enhancement of antibody production has been observed in the mouse system.

Vaccine efficacy studies demonstrated that our F1mutV-PA dual vaccine is highly effective in protection against both anthrax and plague challenges. This has been rigorously tested in three different animal models using (i) multiple challenge formats; sequential challenge and simultaneous challenge with lethal doses of both LeTx and *Y. pestis* CO92, (ii) multiple routes of administration; i.n., intraperitoneal/i.v., and aerosol administration of *Y. pestis* CO92, LeTx, and *B. anthracis* Ames spores, respectively, and (iii) two of the best animal models available for inhalation anthrax (New Zealand White rabbit) and pneumonic plague (Brown Norway rat). Indeed, our studies are the first to demonstrate complete protection of vaccinated animals against simultaneous administration of both anthrax and plague.

Our study represents a new approach to develop a biodefense vaccine that can simultaneously protect against both inhalation anthrax and pneumonic plague. The recombinant F1mutV-PA vaccine is soluble and can be cost-effectively produced in *E. coli* on a large scale. It can be adjuvanted with Alum or another licensed adjuvant using the already established processes in vaccine manufacturing. Indeed, F1mutV-PA adjuvanted with liposomes or Alum–liposomes mixture provided similarly robust immune responses and complete protection against both anthrax and plague (data not shown).

Thus, the bivalent F1mutV-PA anthrax–plague vaccine described here is a strong candidate for human clinical trials to test for safety and to determine optimal antigen dose for eliciting potent and durable antibodies. If successful, it could streamline efforts to stockpile a biodefense vaccine as part of our national preparedness against two of the deadliest bioterror threats, anthrax and plague.

## Materials and Methods

### Construction of Recombinant Plasmids

The *E. coli* expression vector pET28b (EMD Biosciences, Darmstadt, Germany) was used for recombinant plasmid construction. Plasmid pET-F1mutV was constructed in previous studies (26, 29, 52) by fusing V to the C-terminus of mutant F1mut. To construct pET-F1mutV-PA, the *Hin*dIII site (underlined) in the PA was destroyed by overlap extension (SOE) polymerase chain reaction with the primers listed below. The PA fragment was amplified using a 5’- and 3’-end primers containing *Hin*dIII and *Xho*I restriction sites (underlined) at the 5’-end of the primers, respectively. The 5’-end primer also contains a short linker sequence (bolded) that keeps the insert in-frame with the upstream sequence upon cloning. The primer sequences are as follows:

*Hin*dIII Forward: 5′-CCCAAGCTT***CTGCT***GAAGTTAAACAGGAGAACCGGTTATT-3′
Upstream Reverse: 5′-GTGATTAATAAAGCCTCTAATTCTAACAAA-3′
Downstream Forward: 5′-TTTGTTAGAATTAGAGGCTTTATTAATCAC-3′
*Xho*I Reverse: 5′-GCCCTCGAGTTATCCTATCTCATAGCCTTTTTTAG-3′’

The amplified PA fragment was double-digested with *Hin*dIII and *Xho*I and inserted into the pET-F1mutV-Soc (29) that was linearized with the same enzymes. The resulting pET-F1mutV-PA recombinant plasmid contains the PA fragment fused in-frame to the C-terminus of F1mutV with a short linker Glu-Ala-Ser-Ala in between. The final triple antigen construct has the sequence shown in Figure S1A in Supplementary Material. The accuracy of the construct was confirmed by DNA sequencing.

### Purification of Proteins

PA and LF were purified as described previously (53, 54). The *E. coli* BL21-codon plus (DE3)-RIPL cells (Agilent Technologies, Santa Clara, CA, USA) harboring the recombinant plasmid constructed as above were induced with 1 mM Isopropyl β-D-1-thiogalactopyranoside for 2 h at 28°C. Cells were harvested and resuspended in binding buffer (50 mM Tris–HCl pH 8, 300 mM NaCl, and 20 mM imidazole) containing protease inhibitor cocktail (Roche, Indianapolis, IN, USA). Cells were lysed at 1,200 psi using a French press (Aminco, Urbana, IL, USA), and the soluble fractions containing the His-tagged fusion proteins were isolated by centrifugation at 34,000 × *g* for 20 min. Proteins were first subjected to purification by HisTrap column (AKTA-prime, GE Healthcare Bio-Sciences Corp., Piscataway, NJ, USA). Peak fractions containing the desired protein were further purified by size exclusion chromatography on a HiLoad 16/60 Superdex 200 column (AKTA-FPLC, GE Healthcare Bio-Sciences Corp) in a buffer containing 20 mM Tris–HCl pH 8 and 100 mM NaCl. The purified proteins were quantified and stored at −80°C until use. The Endosafe-PTS system (Charles River Laboratories International, Inc., Wilmington, MA, USA) was used to determine the levels of lipopolysaccharide (LPS) contamination in the purified recombinant proteins, and LPS-free preparations were used for animal immunizations.

### Biochemical Functional Analysis of F1mutV-PA

To determine furin protease cleavage sensitivity of F1mutV-PA in comparison with PA, the purified proteins were incubated with different amounts of purified human furin (aa residues 1–604; kindly provided by Dr. Iris Lindberg, University of Maryland Medical School, Baltimore, MD, USA) (55). F1mutV-PA or PA was treated with different molar ratios of protein to furin (200,000:1 to 160:1) in 20 µl buffer containing 50 mM HEPES, pH 7.5, 2 mM CaCl_2_, 0.5 mM EDTA, and 0.2% β-octylglucoside. The reactions were performed at 37°C for 30 min and terminated by adding 2× SDS loading buffer and transferring to a boiling water bath for 5 min. Samples were analyzed by 4–20% gradient SDS-PAGE.

To determine the binding of F1mutV-PA or PA to CMG2 receptor, the purified proteins were incubated with the purified external soluble domain of CMG2 receptor (aa residues 40–218; kindly provided by Dr. Robert Liddington, Sanford-Burnham Medical Research Institute, La Jolla, CA, USA) (56) at room temperature for 30 min. F1mutV-PA or PA was treated with different amounts of CMG2 (molar ratio of protein to CMG2 varied from 4.8:1 to 0.15:1) in 20 µl buffer containing 50 mM HEPES, pH 7.5, 2 mM CaCl_2_, 0.5 mM EDTA, and 0.2% β-octylglucoside. The formation of complexes was analyzed by native PAGE using 4–12% gradient gels (Invitrogen).

To determine the binding of F1mutV-PA or PA to LFn, the N-terminal domain of LF was mixed with the furin-cleaved F1mutV-PA or PA at a molar ratio (protein to LFn) of 1.92:1 to 0.06:1. F1mutV-PA or PA was cleaved by furin as described above using a protein:furin ratio of 160:1. The reactions were performed at room temperature for 30 min in the same buffer as above, and the formation of complexes was evaluated by Native-PAGE using 4–12% gradient gels (Invitrogen). The SDS-PAGE and native gels were stained with Coomassie blue R-250 and Coomassie blue G-250, respectively. The intensity of the bands was quantified using the Image Lab software. Comparisons of function were based on equimolar concentrations of the proteins used for analyses.

### Mouse Immunizations and Challenges

Six- to eight-week-old female Balb/c mice (17–20 g) were purchased from The Jackson Laboratory (Bar Harbor, ME, USA) and randomly grouped and acclimated for 7 days. The purified proteins were adsorbed mixed with Alhydrogel^®^ (Brenntag Biosector, Frederikssund, Denmark) containing 0.19 mg of aluminum per dose. For F1mutV + PA group, the F1mutV and PA antigens were first mixed and then added to equal volume of Alhydrogel. The components were thoroughly mixed to make the final formulation used for immunizations. A total of 25 µg antigen was injected for the F1mutV and PA groups, 25 µg F1mutV plus 25 µg PA for the F1mutV + PA group, and 50 µg F1mutV-PA for the F1mutV-PA group on days 0 and 21 *via* the i.m. route. Control mice received the same amount of Alhydrogel^®^, but without any antigen. Alternate legs were used for each immunization. Blood was collected from each animal by the retro-orbital route on days 0 (prebleeds) and 35 for immunological analyses. In some studies, mice were i.p. challenged first with 1 LD_100_ of LeTx followed by i.n. challenge with 400 LD_50_ (1 LD_50_ = 100 CFU in Balb/c mice) of *Y. pestis* CO92 33 days after LeTx challenge. In other studies, mice were i.p. challenged with 1 LD_100_ of LeTx followed by i.n. challenge with 200 LD_50_ *Y. pestis* CO92 on the same day. Animals were monitored twice daily for mortality and other clinical symptoms.

### Rat Immunizations and Challenges

Five- to six-week-old female Brown Norway rats (50–75 g), purchased from Charles River Laboratories (New Jersey, NJ, USA), were randomized into four groups (nine rats per group) and were acclimated for 7 days before manipulation. The immunogens were formulated and rats were immunized *via* i.m. route as described above for mice. Sera were obtained on day 35 for immunological analyses. The animals were bled by the saphenous vein. Rats were first intranasally challenged on day 42 with ~400 LD_50_ *Y. pestis* CO92 and monitored twice daily for morbidity and mortality over a period of 69 days. The animals that survived were further challenged with 1 LD_100_ LeTx [7.5 µg of each of the toxin components (LF and PA) by the i.v. route] and monitored for another 24 days for morbidity and mortality. In a separate experiment, rats (*n* = 6) were immunized with the immunogen formulations as described above for mice. Two weeks after the boost, rats were challenged simultaneously with 1 LD_100_ of LeTx and 400 LD_50_ *Y. pestis* CO92 as described above.

### Rabbit Immunization and Challenge

The rabbit study was conducted by the Southern Research Institute (Study No: 13538.01.15; Birmingham, AL). A total of 22 New Zealand white rabbits were divided into three groups. Group 1 was vaccinated with F1mutV-PA (50 µg; *n* = 10), while group 2 received PA (25 µg; *n* = 6) alone. Group 3 was naïve control (*n* = 6). Alhydrogel^®^ was used as an adjuvant in groups 1 and 2 (600 μg/rabbit). Control animals (group 3) received the same amount of Alhydrogel^®^ but without any antigen. Rabbits were immunized on day 0 and given a boost on day 14. Sera were collected on days 0 (preimmune), 12, 20, and 42 for immunological analyses. Animals were challenged with 200 LD_50_ of aerosolized *B. anthracis* Ames spores on day 28 and monitored for body weight, body temperature, and mortality until day 42 at which point the remaining animals were euthanized. On days 27, 29–33, and 42, blood samples (~0.2 ml) were collected from the central ear artery into tubes containing sodium polyanethole sulfonate and processed for qualitative microbiological analysis (bacteremia) on the same day. On day 42, the remaining animals were euthanized by an i.v. administration of a barbiturate overdose for tissue collection (brain, liver, lung, and spleen). Tissues were further processed for microbiological and histological analyses.

### Determination of IgG and IgG Subtype Antibodies

Antibody titers were determined by ELISA as described previously (26). Briefly, 96-well plates were coated with 100 ng/well of purified F1mutV or PA antigen at 4°C overnight. Following blocking and washing, serum samples were serially diluted and incubated with the affixed antigens for 1 h at 37°C. Following five washes, horseradish peroxidase (HRP)-conjugated goat anti-mouse IgG secondary antibodies (Invitrogen, Camarillo, CA, USA) were added to the wells at a dilution of 1:5,000. After incubation for 1 h at 37°C, unbound antibodies were removed and the wells were washed five times with PBS-T (PBS containing 0.05% Tween-20). Hundred microliters of TMB Microwell Peroxidase Substrate solution (KPL, Gaithersburg, MD, USA) was added to each well. Following 3 min incubation at room temperature to develop the color, the reaction was quenched by the addition of the same volume of TMB BlueSTOP^TM^ solution (KPL) and absorbance was read at 650 nm using an ELISA reader (Molecular Devices, Sunnyvale, CA, USA). For rat IgG, HRP-conjugated goat anti-rat IgG (KPL, Gaithersburg, MD, USA) was used as the secondary antibody. For mouse or rat IgG subtypes, HRP-conjugated goat anti-mouse or anti-rat IgG1 or IgG2a secondary antibodies (Abcam, Cambridge, MA, USA) were used. For rabbit anti-PA IgG titers, plates were coated with PA and affinity-purified rabbit anti-PA polyclonal antibody was used to generate a standard curve, from which the sample anti-PA IgG concentrations (ng/mL) were determined. Samples were initially at 1:200; additional dilutions were performed as necessary to ensure that values could be determined from the standard curve.

### Anthrax LeTx Neutralization Assay (TNA)

Anthrax LeTx neutralization assay (TNA) was performed as described previously (57). Briefly, PA and LF (200 ng/ml each) were prepared in Dulbecco’s modified Eagle’s medium, and sera were diluted serially into the toxin mixture and incubated for 1 h at 37°C. Toxin–serum mixtures were transferred to RAW 264.7 macrophage cells grown to confluence in 96-well plates and incubated for 5 h, and cell viability assessed by incubation with 3-(4,5-dimethylthiazo-2-yl)-2,5-diphenyltetrazolium bromide (Sigma, St. Louis, MO, USA) at a final concentration of 0.5 mg/ml for 30 min. An insoluble pigment (formazan) produced by living cells was dissolved by adding a solution containing 0.5% SDS, 25 mM HCl, and 90% isopropanol, and the optical density (570 nm) measured to assess viability. The effective serum concentration inducing 50% neutralization (EC_50_) was calculated with Prism software (Graphpad Software, Inc., San Diego, CA, USA).

### Live Animal Imaging

Depending on the experiment (described above), 2 or 3 days after challenge with *Y. pestis* CO92-luciferase strain, the animals were imaged by using an IVIS 200 bioluminescence and fluorescence whole-body imaging workstation (Caliper Corp., Alameda, CA, USA) in the ABSL-3 facility at UTMB following light anesthesia under isoflurane.

### Statistical Analyses

Results are expressed as mean ± SD. Statistical comparisons among different groups were evaluated by analysis of variance. The animal mortality data were analyzed by the Kaplan–Meier survival estimate. A value of *p* < 0.05 was considered statistically significant.

## Ethics Statement

This study was conducted in accordance with the Guide for the Care and Use of Laboratory Animals recommended by the National Institutes of Health. All animal experiments were performed according to the protocols approved by the Institutional Animal Care and Use Committees of the University of Texas Medical Branch, Galveston, TX, USA (Office of Laboratory Animal Welfare assurance number: A3314-01), The Catholic University of America, Washington, DC, USA (Office of Laboratory Animal Welfare assurance number: A4431-01), and Southern Research Institute, Birmingham, AL, USA (Office of Laboratory Animal Welfare assurance number: A3046-01).

## Author Contributions

PT, AC, and VR designed the experiments. PT, MMa, JZ, MMo, MK, EF, JA, and WL performed the experiments. PT, MMa, JZ, MMo, MK, SL, AC, and VR analyzed the data. PT, AC, and VR wrote the manuscript. VR directed the project.

## Conflict of Interest Statement

The authors declare that the research was conducted in the absence of any commercial or financial relationships that could be construed as a potential conflict of interest. The reviewer EA and handling Editor declared their shared affiliation, and the handling Editor states that the process nevertheless met the standards of a fair and objective review.

## Supplementary Material

Figure S1 Purification and character of F1mut1-protective antigen (PA). **(A)** The amino acid sequence of recombinant F1mutV-PA triple antigen. The F1mut is shown in green; V and PA are shown in purple and blue, respectively; The His-tag and linkers are shown in orange and black individually. The N-terminal β-strand (residues 1–14) of F1, which was switched to C-terminus, is highlighted with underline. The 7 amino acids (residues 15–21), which was repeated, were italicized. **(B)** Purification of the F1mut1-PA. The F1mut1-V recombinant protein was purified from the cell-free lysates by HisTrap affinity chromatography followed by Hi-load 16/60 Superdex 200 gel filtration. The figure shows the elution profile on the Superdex 200 column and the inset shows the purity of F1mut1-PA after SDS-PAGE and Coomassie blue staining of the pooled peak fractions.

Figure S2 Body weight changes in male **(A)** and female **(B)** rabbits, and body temperature changes in male **(C)** and female **(D)** rabbits after *Bacillus anthracis* challenge (200 LD_50_, aerosol). Animals were immunized (intramuscular) according to Figures 8A,B and challenged (aerosol) with 200 LD_50_ *B. anthracis* 2 weeks after last immunization. The rabbits were monitored daily for body weight and body temperature.
