# Supplementary material for: A Bivalent Anthrax–Plague Vaccine That Can Protect against Two Tier-1 Bioterror Pathogens, Bacillus anthracis and Yersinia pestis
Source: Front Immunol. 2017 Jun 26;8:687. doi: 10.3389/fimmu.2017.00687 (PMC5483451; doi:10.3389/fimmu.2017.00687)
Supplement: Supplementary file 1 [file Table_1.DOCX]

**Supplementary Table 1. Summary of Challenge Experiments**

| **Animal models** | | **Challenge agent** | **Challenge dose (route)** | **Schedule** |
| --- | --- | --- | --- | --- |
| **Mouse** | sequential challenge | LeTx and  *Y. pestis* CO92 | 1 LD_100_ of LeTx (i.p.) and 400 LD_50_ of *Y. pestis* (i.n.) | Challenge with LeTx on day zero followed by *Y. pestis* challenge on day 33 |
|  | simultaneous challenge | LeTx and  *Y. pestis* CO92 | 1 LD_100_ of LeTx (i.p.) and 200 LD_50_ of *Y. pestis* (i.n.) | Simultaneous challenge with LeTx and *Y. pestis* on day zero |
| **Rat** | sequential challenge | LeTx and  *Y. pestis* CO92 | 400 LD_50_ of *Y. pestis* (i.n.) and 1 LD_100_ of LeTx (i.v.) | Challenge with *Y. pestis* on day zero followed by LeTx challenge on day 70 |
|  | simultaneous challenge | LeTx and  *Y. pestis* CO92 | 1 LD_100_ of LeTx (i.v.) and 400 LD_50_ of *Y. pestis* (i.n.) | Simultaneous challenge with LeTx and *Y. pestis* on day zero |
| **Rabbit** | | *B. anthracis* Ames spores | 200 LD_50_ of aerosolized *B. anthracis* Ames spores (i.n.) | challenge with aerosolized *B. anthracis* Ames spores on day zero |
