# Supplementary material for: A Bivalent Anthrax–Plague Vaccine That Can Protect against Two Tier-1 Bioterror Pathogens, Bacillus anthracis and Yersinia pestis
Source: Front Immunol. 2017 Jun 26;8:687. doi: 10.3389/fimmu.2017.00687 (PMC5483451; doi:10.3389/fimmu.2017.00687)
Supplement: Supplementary file 2 [file Table_2.DOCX]

**Supplementary Table 1. Summary of Qualitative Bacteremia in Blood**

| **Animal ID** | | **Day 27** | **Day 29** | **Day 30** | **Day 31** | **Day 32** | **Day 33** | **Day 42** |
| --- | --- | --- | --- | --- | --- | --- | --- | --- |
| Group 1  F1mutV-PA  (Male) | 2M14117 | **-** | **-** | **-** | **-** |  | **-** | **-** |
|  | 2M14122 | **-** | **-** | **-** | **-** |  | **-** | **-** |
|  | 2M14120 | **-** | **-** | **-** | **-** |  | **-** | **-** |
|  | 2M14106 | **-** | **-** | **-** | **-** |  | **-** | **-** |
|  | 2M14111 | **-** | **-** | **-** | **-** |  | **-** | **-** |
| Group 1 F1mutV-PA  (Female) | 2F14123 | **-** | **-** | **-** | **-** |  | **-** | **-** |
|  | 2F14124 | **-** | **-** | **-** | **-** |  | **-** | **-** |
|  | 2F14129 | **-** | **-** | **-** | **-** |  | **-** | **-** |
|  | 2F141134 | **-** | **-** | **-** | **-** |  | **-** | **-** |
|  | 2F14136 | **-** | **-** | **-** | **-** |  | **-** | **-** |
| Group 2  PA  (Male) | 3M14110 | **-** | **-** | **-** | **-** |  | **-** | **-** |
|  | 3M14115 | **-** | **-** | **-** | **-** |  | **-** | **-** |
|  | 3M14118 | **-** | **-** | **-** | **-** |  | **-** | **-** |
| Group 2  PA  (Female) | 3F14127 | **-** | **-** | **-** | **-** |  | **-** | **-** |
|  | 3F14139 | **-** | **-** | **-** | **-** |  | **-** | **-** |
|  | 3F14141 | **-** | **-** | **-** | **-** |  | **-** | **-** |
| Group 3  PBS control  (Male) | 4M14107 | **-** | **-** | **+** | **+** |  |  |  |
|  | 4M14112 | **-** | **-** | **+** |  |  |  |  |
|  | 4M14131 | **-** | **-** | **-** | **+** | **+** |  |  |
| Group 3  PBS control  (Female) | 4F14105 | **-** | **-** | **-** | **+** |  |  |  |
|  | 4F14125 | **-** | **-** | **+** | **+** |  |  |  |
|  | 4F14140 | **-** | **-** | **+** |  |  |  |  |

Blood samples for bacteremia analysis were drawn before the challenge on day 27 (baseline), on days 29-33 (1-5 days post-exposure), and on day 42. Vaccinated animals (Group 1 and 2) never developed bacteremia whereas all unvaccinated control animals (Group 3) became positive for bacteremia before they succumbed to the disease.
