# Supplementary material for: A Bivalent Anthrax–Plague Vaccine That Can Protect against Two Tier-1 Bioterror Pathogens, Bacillus anthracis and Yersinia pestis
Source: Front Immunol. 2017 Jun 26;8:687. doi: 10.3389/fimmu.2017.00687 (PMC5483451; doi:10.3389/fimmu.2017.00687)
Supplement: Supplementary file 3 [file Table_3.DOCX]

| **Animal ID** | | **Brain (CFU/g)** | **Liver (CFU/g)** | **Lung (CFU/g)** | **Spleen (CFU/g)** |
| --- | --- | --- | --- | --- | --- |
| Group 1  F1mutV-PA  (Male) | 2M 14117 | 0.0E+00 | 0.0E+00 | 0.0E+00 | 0.0E+00 |
|  | 2M 14122 | 0.0E+00 | 0.0E+00 | 0.0E+00 | 0.0E+00 |
|  | 2M 14120 | 0.0E+00 | 0.0E+00 | 0.0E+00 | 0.0E+00 |
|  | 2M 14106 | 0.0E+00 | 0.0E+00 | 0.0E+00 | 0.0E+00 |
|  | 2M 14111 | 0.0E+00 | 0.0E+00 | 0.0E+00 | 0.0E+00 |
| Group 1  F1mutV-PA  (Female) | 2F 14123 | 0.0E+00 | 0.0E+00 | 0.0E+00 | 0.0E+00 |
|  | 2F 14129 | 0.0E+00 | 0.0E+00 | 0.0E+00 | 0.0E+00 |
|  | 2F 14124 | 0.0E+00 | 0.0E+00 | 0.0E+00 | 0.0E+00 |
|  | 2F 14136 | 0.0E+00 | 0.0E+00 | 0.0E+00 | 0.0E+00 |
|  | 2F 14134 | 0.0E+00 | 0.0E+00 | 0.0E+00 | 0.0E+00 |
| Group 2  PA  (Male) | 3M 14110 | 0.0E+00 | 0.0E+00 | 0.0E+00 | 0.0E+00 |
|  | 3M 14115 | 0.0E+00 | 0.0E+00 | 0.0E+00 | 0.0E+00 |
|  | 3M 14118 | 0.0E+00 | 0.0E+00 | 0.0E+00 | 0.0E+00 |
| Group 2  PA  (Female) | 3F 14127 | 0.0E+00 | 0.0E+00 | 0.0E+00 | 0.0E+00 |
|  | 3F 14141 | 0.0E+00 | 0.0E+00 | 0.0E+00 | 0.0E+00 |
|  | 3F 14139 | 0.0E+00 | 0.0E+00 | 0.0E+00 | 0.0E+00 |
| Group 3  PBS control  (Male) | 4M 14112 | 6.4E+06 | 7.5E+06 | 9.9E+06 | 7.9E+06 |
|  | 4M 14107 | 9.8E+05 | 2.9E+06 | 9.1E+08 | 1.4E+08 |
|  | 4M 14105 | 2.0E+06 | 5.6E+07 | 1.4E+09 | 2.5E+09 |
| Group 3  PBS control  (Female) | 4F 14131 | 1.0E+07 | 0.0E+00 | 4.6E+06 | 1.9E+06 |
|  | 4F 14125 | 2.4E+06 | 3.9E+07 | 8.0E+08 | 2.7E+08 |
|  | 4F 14140 | 7.9E+06 | 4.9E+07 | 4.8E+07 | 6.2E+06 |

**Supplementary Table 2. Individual Chart of Bacterial Load of Tissue Samples**

To determine the bacterial load of internal organs, post-mortem collection of specimens was performed after scheduled euthanasia of surviving animals on study day 42 (Group 1 and 2) or after animals died due to the anthrax exposure (Group 3). All vaccinated animals from Group 1 and 2 had cleared the agent from the lungs and did not have any bacteria in brain, liver, or spleen. Tissue samples collected from unvaccinated control animals (Group 3) had very high bacterial titers in all the organs tested indicative of systemic anthrax infection.
