# Supplementary figures and images for: A Bivalent Anthrax–Plague Vaccine That Can Protect against Two Tier-1 Bioterror Pathogens, Bacillus anthracis and Yersinia pestis
Source: Front Immunol. 2017 Jun 26;8:687. doi: 10.3389/fimmu.2017.00687 (PMC5483451; doi:10.3389/fimmu.2017.00687)

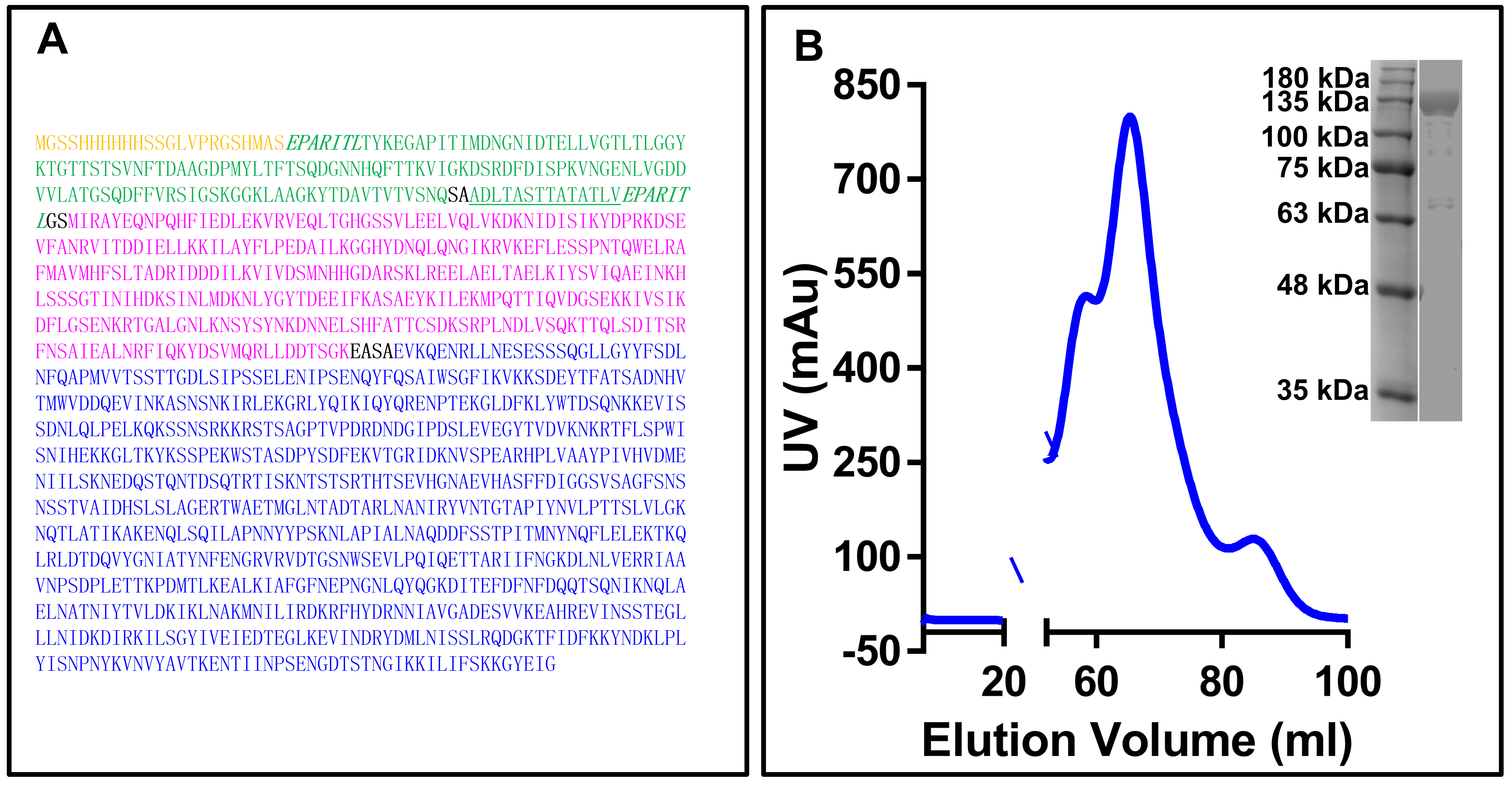

Supplement: Figure S1 — Purification and character of F1mut1-protective antigen (PA). (A) The amino acid sequence of recombinant F1mutV-PA triple antigen. The F1mut is shown in green; V and PA are shown in purple and blue, respectively; The His-tag and linkers are shown in orange and black individually. The N-terminal β-strand (residues 1–14) of F1, which was switched to C-terminus, is highlighted with underline. The 7 amino acids (residues 15–21), which was repeated, were italicized. (B) Purification of the F1mut1-PA. The F1mut1-V recombinant protein was purified from the cell-free lysates by HisTrap affinity chromatography followed by Hi-load 16/60 Superdex 200 gel filtration. The figure shows the elution profile on the Superdex 200 column and the inset shows the purity of F1mut1-PA after SDS-PAGE and Coomassie blue staining of the pooled peak fractions. [file Image_1.TIF]

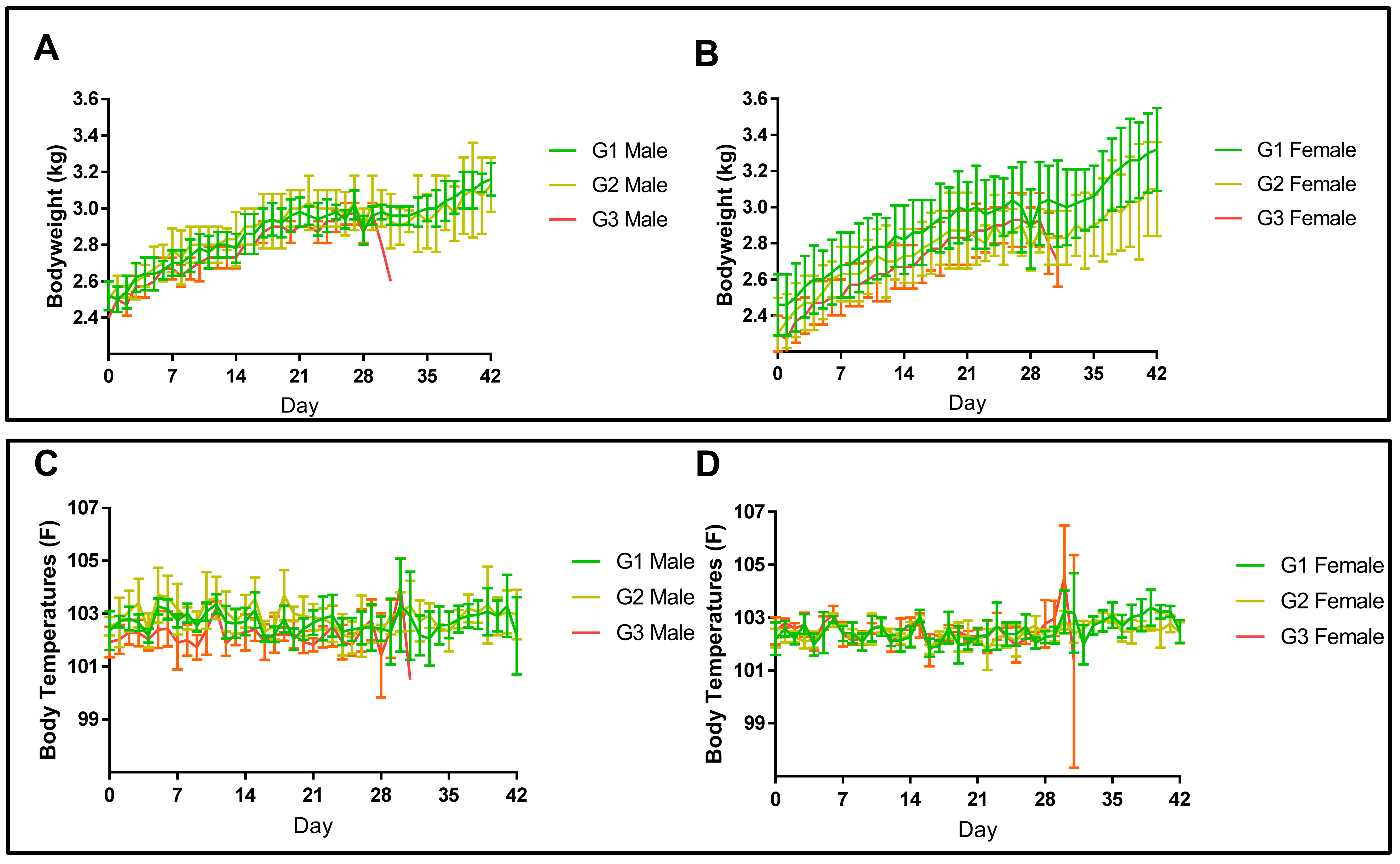

Supplement: Figure S2 — Body weight changes in male (A) and female (B) rabbits, and body temperature changes in male (C) and female (D) rabbits after Bacillus anthracis challenge (200 LD50, aerosol). Animals were immunized (intramuscular) according to Figures 8A,B and challenged (aerosol) with 200 LD50 B. anthracis 2 weeks after last immunization. The rabbits were monitored daily for body weight and body temperature. [file Image_2.TIF]
